# Dopamine downregulation in novel rodent models useful for the study of postpartum depression

**DOI:** 10.3389/fnbeh.2022.1065558

**Published:** 2022-12-22

**Authors:** Millie Rincón-Cortés, Anthony A. Grace

**Affiliations:** ^1^Department of Neuroscience, School of Behavioral and Brain Sciences, University of Texas at Dallas, Richardson, TX, United States; ^2^Departments of Neuroscience, Psychiatry and Psychology, University of Pittsburgh, Pittsburgh, PA, United States

**Keywords:** animal models-rodent, postpartum depression, maternal behavior, reward, adversity, dopamine, ventral tegmental area

## Abstract

Postpartum depression (PPD) is the most common psychiatric disorder following childbirth and is characterized by maternal mood disturbances, impaired maternal responses, and disrupted caregiving- all of which negatively impact offspring development. Since PPD has detrimental consequences for both mother and child, clinical and preclinical research has focused on identifying brain changes associated with this disorder. In humans, PPD is linked to dysregulated mesolimbic dopamine (DA) system function and altered neural responses (i.e., decreased reward-related activity) to infant-related cues, which are considered hallmark features of PPD. In accordance, rodent models employing translational risk factors useful for the study of PPD have demonstrated alterations in mesolimbic DA system structure and function, and these changes are reviewed here. We also present two novel rodent models based on postpartum adversity exposure (i.e., pup removal, scarcity-adversity) which result in PPD-relevant behavioral changes (e.g., disrupted mother-infant interactions, deficits in maternal behavior, depressive-like phenotypes) and attenuated ventral tegmental area (VTA) DA neuron activity consistent with a hypodopaminergic state. Furthermore, we highlight open questions and future directions for these rodent models. In sum, human and rodent studies converge in showing blunted mesolimbic DA function (i.e., DA downregulation) in PPD. We propose that reduced activity of VTA DA neurons, resulting in downregulation of the mesolimbic DA system, interferes with reward-related processes necessary for maternal motivation and responsiveness. Thus, the mesolimbic DA system may constitute a therapeutic target for ameliorating reward-related deficits in PPD.

## 1. Introduction

Postpartum depression (PPD) is the most common psychiatric disorder following childbirth in women (Thurgood et al., 2009; Austin et al., 2010). Core symptoms of PPD include depressed mood, poor social adjustment following parturition (i.e., delivery), and loss of interest or pleasure (anhedonia; Pearlstein et al., 2009; Thurgood et al., 2009; Post and Leuner, 2019). In PPD, anhedonia primarily impacts the social domain and involves loss of interest in the infant and infant-related stimuli, which may cause difficulties in adjusting to new activities characteristic of the new role as a mother (i.e., caregiving; Eid et al., 2019; Post and Leuner, 2019). Mothers with PPD often show hostility, less affection, lower infant engagement, and reduced responsiveness/sensitivity to their baby’s needs (Lovejoy et al., 2000; Pearlstein et al., 2009; Field, 2010). Thus, PPD places children at risk for impaired behavioral, cognitive, and emotional development as well as later life psychopathology (Beck, 1998; Grace et al., 2003; Murray et al., 2011; Sanger et al., 2015; Netsi et al., 2018). The widespread prevalence of PPD and its negative consequences on both mother and child underscores the importance of understanding the neurobiological underpinnings of PPD, which may aid in guiding treatments and developing strategies for improving functional deficits in caregiving.

One neural system implicated in PPD is the mesolimbic dopamine (DA) system, which is critically involved in reward-related and motivated maternal behaviors in both humans and rodents (Robinson et al., 2011; Olazabal et al., 2013; Nephew et al., 2015; Post and Leuner, 2019; Rincón-Cortés and Grace, 2020a). Activation of the mesolimbic DA system, which originates in the ventral tegmental area (VTA) and consists of dopaminergic projections to cortical and limbic regions, is part of a central mechanism that drives infant-seeking behavior and strengthens the mother-infant bond (Fleming et al., 1994; Rincón-Cortés and Grace, 2020a). The VTA is part of the maternal caregiving network, which refers to brain areas/neural systems important for maternal behaviors that are activated or inhibited in response to offspring, including VTA targets such as the basolateral amygdala (BLA), nucleus accumbens (NAc), and prefrontal cortex (PFC; Pereira and Morrell, 2011; Pawluski et al., 2017; Rincón-Cortés and Grace, 2020a). The functional integrity of the VTA and mesolimbic DA signaling is causally linked to the adequate expression of maternal behavior (Numan, 2007; Pereira and Morrell, 2011; Rincón-Cortés and Grace, 2020a).

Human and rodent research suggest that PPD is mediated by deficiencies within the brain’s reward pathway (Nephew et al., 2015; Post and Leuner, 2019). Neuroimaging studies in humans have identified atypical mesolimbic DA system function in PPD (Moses-Kolko et al., 2014; Duan et al., 2017). For example, mothers with PPD exhibit reduced ventral striatal activation in response to monetary rewards and positive words, which is linked to greater PPD symptomatology (Silverman et al., 2007; Moses-Kolko et al., 2011). This is significant given that hypoactivation of the ventral striatum (e.g., NAc, caudate, putamen) in response to rewarding and/or pleasant stimuli is thought to mediate reductions in reward-seeking behaviors, which is a hallmark of depression (Keedwell et al., 2005; Epstein et al., 2006; Dunlop and Nemeroff, 2007; Pizzagalli et al., 2009). Depressed mothers also exhibit diminished ventral striatal responses to infant-related stimuli (Laurent and Ablow, 2012), which is opposite to the robust ventral striatal and VTA response shown by control mothers (Bartels and Zeki, 2004; Noriuchi et al., 2008; Strathearn et al., 2008). These findings suggest attenuated mesolimbic DA system function in PPD, which could contribute to decreased motivation and goal-directed behaviors. Consistent with this notion, this perspective provides an overview of rodent models relevant to PPD in which mesolimbic DA dysfunction (namely reduced DA activity) has been identified. We also present two novel rodent models based on postpartum adversity that are characterized by hypoactivity of VTA DA neurons, which is thought to reduce mesolimbic DA system activation, and is aligned with clinical and preclinical data suggesting dampened maternal motivation and blunted reward-related brain activity in PPD. We propose that mesolimbic DA system downregulation, driven by attenuated VTA DA activity, interferes with reward-related processes necessary for motivated maternal behaviors and may represent a therapeutic target for ameliorating reward-related deficits in PPD.

## 2. Mesolimbic Dopamine System Dysregulation in Rodent Models Relevant to PPD

Mesolimbic DA system alterations have been reported in several rodent models relevant to PPD, including stress-, genetic-, and diet-based models. Stress-based rodent models commonly employ stress exposure during pregnancy (i.e., gestational stress), which increases the risk for PPD in humans (Robertson et al., 2004; Hillerer et al., 2012; Qiu et al., 2020; Mir et al., 2022). Rat dams exposed to chronic gestational stress display impaired maternal care (i.e., more time away from the nest, reduced arch-back nursing) as well as increased passive coping behavior (i.e., immobility) in the forced swim test (FST) during the early postpartum (Smith et al., 2004; Haim et al., 2014; Leuner et al., 2014). Dams exposed to chronic gestational stress also exhibit altered structural plasticity within the NAc, which receives dopaminergic innervation from the VTA and is implicated in PPD (Haim et al., 2014; Post and Leuner, 2019). Specifically, these dams had reductions in dendritic length, branching, and spine density on medium spiny neurons in the NAc shell but not the NAc core (Haim et al., 2014), which is consistent with prior findings suggesting greater sensitivity of the NAc shell to stress (Kalivas and Duffy, 1995; Wu et al., 1999). Thus, gestational stress induces morphological and structural changes within the mesolimbic DA system that may impair DA signaling and reward-related processes involving the primary reward circuit (i.e., VTA-NAc projection). Furthermore, postpartum administration of the selective serotonin reuptake inhibitor (SSRI) citalopram, which restores functionality in mothers with PPD (Misri et al., 2012), reversed the behavioral and structural changes induced by gestational stress exposure in rat dams (Haim et al., 2016). These findings show that the behavioral and NAc changes observed in dams following chronic gestational stress are sensitive to antidepressant administration, suggesting they are reflective of a depressive-like state.

Dopaminergic dysfunction has also been found in genetic-based PPD models, including the selectively bred Flinders Sensitive Line (FSL) and Wistar-Kyoto (WKY) rats (Overstreet et al., 2005; Millard et al., 2020). FSL dams exhibit abnormal maternal behaviors such as reduced pup licking and contact time, reduced NAc DA levels while interacting with pups as measured through microdialysis, no place preference conditioning to pups, and higher levels of FST immobility compared to Sprague-Dawley (SD, control) rats (Lavi-Avnon et al., 2005a,b, 2008). Taken together, these data suggest a lower rewarding value of pups and blunted pup-elicited DA responses in FSL dams. WKY dams exhibit severe deficits in maternal behaviors during a 30-min test following a mild stressor: they made fewer retrievals and repositioning of pups into the nest and displayed reduced licking compared to SD dams, with this difference being more pronounced in the early postpartum (Winokur et al., 2019). WKY dams also took longer to hover over pups and nurse. With regards to DA function, early postpartum WKY dams had lower DA levels within striatal areas and the medial preoptic area, as well as higher DA turnover rates in these structures compared to SD dams (Winokur et al., 2019). Collectively, these data suggest that disturbances in parenting and caregiving behaviors in FSL and WKY dams are associated with alterations in DA levels within various mesolimbic brain regions, suggesting central mesolimbic DA system dysfunction. Preclinical studies conducted in mice have used the BALB/c mouse strain as a genetic-based model useful for the study of PPD. Compared to C57BL/6 mice, BALB/c mice exhibit affective dysregulation (i.e., increased anxiety-like and depressive-like behaviors) and poor mothering (i.e., lower pup licking and grooming; Tarantino et al., 2011). Postpartum BALB/c mice also exhibit reduced mobility time in the FST, which was interpreted as increased immobility, and lower levels of striatal DA compared to C57BL/6 mice (Avraham et al., 2017). The findings observed in BALB/c mouse dams are consistent with those observed in WKY and FSL rat dams.

Finally, changes in depressive-like behaviors and DA system function have also been found in mouse dams that were fed a Western diet (high in fat and/or branched-chain amino acids; Bolton et al., 2017). These dams exhibited increased FST immobility and a significant reduction in DA D2 receptor expression in the hippocampus, which receives dopaminergic innervation from the VTA, as well as reductions in DA metabolites in the hippocampus and PFC during the early postpartum (Bolton et al., 2017). In sum, preclinical studies using distinct approaches (i.e., gestational stress, genetic, diet-based models) recapitulate key features of PPD such as negative maternal affect, disrupted caregiving, and mesolimbic DA dysregulation.

## 3. DA Deficits in Novel Rodent Models Useful for The Study PPD Based on Postpartum Adversity

Postpartum adversity is a strong predictor for the emergence of PPD in humans (O’Hara, 2009; Yim et al., 2015), and a translational risk factor employed in PPD-relevant rodent models (Perani and Slattery, 2014; Li and Chou, 2016; Mir et al., 2022). Below, we review two novel rodent models useful for the study of PPD that are based on creating adverse maternal environments during the early postpartum. The first model consists of manipulating the dam’s social environment by permanently removing pups (Pawluski et al., 2009), which represents an enduring disruption of the mother-infant attachment bond- the strongest social bond in maternal mammals (Bowlby, 1982). The second model consists of manipulating the dam’s physical environment by reducing the amount of bedding material within the dam’s home cage (i.e., resource scarcity; Ivy et al., 2008). We have recently shown that both models converge in disrupting a variety of behaviors in the dam, including maternal and depression-relevant behaviors, and attenuating mesolimbic DA system function (Rincón-Cortés and Grace, 2021, 2022).

### 3.1 Permanent pup removal

A novel rodent model useful for studying behavioral and dopaminergic changes relevant to PPD consists of permanently removing pups from the rat dam’s home cage within 24 h of giving birth. This model is consistent with human literature showing that the emotional impact of losing a child often results in prolonged grief disorder, which in many cases is comorbid with depression (Demarchi et al., 2021). Indeed, disruption or loss of mother-infant attachment bonds is a strong predictor of increased negative affect and elevated depression symptoms in human mothers (Vance et al., 1995; Crouch, 2002; Badenhorst and Hughes, 2007). For example, bereaved women had nearly 4-fold higher odds of a positive screen for depression (Gold et al., 2016). Because we cannot measure grief in rodents, we focus on the increased depression-related phenotypes resulting from permanent pup removal. Permanent pup removal increases passive FST coping responses (i.e., immobility) and reduces social motivation during the late postpartum period in rat dams (Pawluski et al., 2009; Rincón-Cortés and Grace, 2021). These behavioral effects overlap with those observed in female rodent models relevant to depression, including stress-based, pharmacologically-induced and genetic models (Iniguez et al., 2018; Newman et al., 2019; Lima et al., 2022), as well as in PPD rodent models employed during the postpartum (Perani and Slattery, 2014; Li and Chou, 2016; Qiu et al., 2020; Mir et al., 2022).

In terms of mesolimbic DA function, permanent pup removal results in a long-lasting attenuation of VTA DA neuron activity, as indexed by a reduction in the number of active DA neurons ~3 weeks after pup removal (Rincón-Cortés and Grace, 2021). Moreover, this reduction in VTA DA neuron activity was correlated with reduced social sniff time in dams that had pups removed. This is significant because social interaction is driven by activation of the mesolimbic reward circuitry, and specifically increased DA signaling from the VTA into the NAc (Gunaydin et al., 2014). Within this context, a decrease in VTA DA neuron activity would be expected to diminish stimulus-driven DA neuron responses, leading to attenuated mesolimbic DA system activation and blunted reward-related responses, including a reduced social approach (Grace, 2016). Importantly, the enduring effects of permanent pup removal are specific to pup loss from postpartum days (PD) 1–21, as a 3-week social isolation window did not impact social approach, FST immobility, or DA function in adult virgin females (Rincón-Cortés and Grace, 2021). In sum, the presence of pups during the postpartum period appears to be important for establishing adequate levels of maternal affect and mesolimbic DA system activity, whereas the lack of pup presence can trigger a long-lasting negative affect (i.e., depressive-like) state associated with mesolimbic DA downregulation. For instance, permanent pup removal induces increased passive coping in the FST, social withdrawal, and DA downregulation (i.e., reduced VTA DA activity; Rincón-Cortés and Grace, 2021), which are depression-related outcomes. Moreover, these behavioral and DA effects overlap with those commonly observed in rodent models of depression based on chronic stress exposure (Tye et al., 2013; Chang and Grace, 2014; Kaufling, 2019). Yet, the duration of these behavioral and DA effects is unknown. Future studies assessing whether these changes persist throughout future pregnancies and whether they are sensitive to antidepressant administration are needed.

### 3.2 Postpartum scarcity-adversity

Another novel rodent model useful for studying stress-induced neurobehavioral adaptations relevant to PPD consists of creating an impoverished postpartum environment by providing the dam with limited bedding and nesting (LBN) materials from PD 2 to 9 (Rincón-Cortés and Grace, 2022). This procedure increases corticosterone (CORT) levels in the rat dam (Ivy et al., 2008), impairs motivated maternal behavior, and disrupts mother-infant interactions (Rincón-Cortés and Grace, 2022). Specifically, postpartum scarcity-adversity increased the percentage of negative pup-directed behaviors including stepping, dragging, improper transport, and shoving of pups (Rincón-Cortés and Grace, 2022). LBN dams also exhibited longer pup retrieval latencies, suggesting impaired goal-directed maternal responses, as well as passive stress-coping responses (i.e., increased FST immobility). Thus, LBN exposure in rats mimics the effects of a stressful environment in increasing risk for PPD and potentiating negative caregiving patterns (e.g., maltreatment, neglect) similar to what is observed in human mothers (Webster-Stratton, 1990; Hall et al., 1998; Cadzow et al., 1999; Field, 2010; Goyal et al., 2010; Payne and Maguire, 2019).

Dams exhibiting disrupted mother-infant interactions and impaired maternal motivation also had reduced VTA DA neuron population activity (i.e., lower numbers of active DA neurons) compared to control dams at PD 9–10, but not at PD 20–21 (~1.5 weeks after cessation of LBN). This decrease in the number of active DA neurons is proposed to attenuate the ability of DA neurons to respond to reward-related stimuli in a behaviorally salient phasic manner (Grace, 2016). Our findings suggest that the VTA DA decrease is linked to the presence of environmental adversity (as effects are only seen during LBN) and can recover once dams are restored to normal home cage environments. Based on these data, we propose that compromised (i.e., reduced) activity of VTA DA neurons induced by scarcity-adversity likely contributes to postpartum negative affect (i.e., depressive-like behavior) and interferes with reward-related processes necessary for motivated maternal behavior. Future studies should determine when VTA DA deficits emerge in LBN dams and examine a functional temporal link between adversity-induced changes in maternal behaviors and VTA DA neuron activity in real-time. For example, do LBN dams exhibit blunted mesolimbic DA activity during the expression of maladaptive maternal behaviors and/or in response to pups? In sum, postpartum scarcity-adversity disrupts mother-infant attachment, impairs maternal motivation, increases negative affect-related behaviors, and induces mesolimbic DA downregulation in rat dams—all which are consistent with PPD symptoms in humans.

### 3.3 DA deficits in novel rodent models of PPD based on postpartum adversity overlap with those observed in rodent models of chronic stress relevant to depression

Importantly, the behavioral and dopaminergic effects observed in dams that underwent postpartum adversity (i.e., pup removal, LBN) overlap with those observed in rodent models of chronic stress relevant to depression (Belujon and Grace, 2017; Douma and de Kloet, 2019; Rincón-Cortés and Grace, 2020b). For instance, adult male and female rats exposed to chronic mild stress (CMS) exhibit increased FST immobility (i.e., depression-related behavior) as well as hypoactivity of VTA DA neurons (i.e., DA downregulation = reduced numbers of active DA cells; Chang and Grace, 2014; Moreines et al., 2017; Rincón-Cortés and Grace, 2017; Neves and Grace, 2019). This reduction in VTA DA activity is significant given that it is causally linked to the expression of depressive-like behaviors in rodents (Tye et al., 2013). Increasing the activity of VTA DA neurons *via* optogenetic stimulation increases FST escape-related behaviors (i.e., kicking) in rats and rescues stress-induced depression-related behavioral phenotypes (Tye et al., 2013). Therefore, increasing VTA DA activity in dams undergoing pup removal or scarcity-adversity may reverse the increased FST immobility induced by both models. Since VTA DA neurons (and specifically increases in VTA DA activity) are critically involved in motivated behaviors, including social approach and pup retrieval (Gunaydin et al., 2014; Fang et al., 2018), this manipulation could also potentially rescue blunted social motivation in dams that underwent pup removal and/or impaired pup retrieval in LBN dams.

With regards to afferent regulation of VTA DA function, the attenuation in VTA population activity induced by chronic stress is driven by BLA hyperexcitability and specifically increased activity within the BLA-ventral pallidum (VP) pathway (Chang and Grace, 2014; Neves and Grace, 2019). Stress-induced activation of this pathway inhibits VTA activity, whereas inhibition of the BLA or the VP prevents the stress-induced decrease (Chang and Grace, 2014). Since the BLA is a stress-sensitive structure that regulates VTA DA function (Belujon and Grace, 2015) and is critical for motivated maternal behaviors (Numan et al., 2010), we propose that VTA DA neuron hypoactivity in dams that underwent pup removal and LBN may be driven by BLA hyperactivity and increased activity within the BLA-VP pathway, thereby resulting in impaired maternal motivation. If this is the case, normalizing activity within this pathway may reverse the behavioral and VTA DA changes induced by postpartum adversity. However, the impact of pup removal or scarcity-adversity on BLA activity in the dam has yet to be assessed. This information would be helpful for determining whether similar structures/pathways drive VTA DA neuron hypoactivity in both sexes (and across reproductive conditions) and uncovering ways to reverse DA downregulation in PPD rodent models based on postpartum adversity.

## 4. Discussion

Clinical and preclinical studies show that PPD is associated with dysfunction within the brain’s reward pathway- the mesolimbic DA system. In rodent models, these alterations occur across multiple levels and include structural and functional plasticity, as well as basal and pup-evoked changes in concentrations of DA metabolites. Mesolimbic DA system changes in PPD rodent models are summarized in Table 1. These adaptations are thought to interfere with reward-related processes necessary for motivated maternal behaviors and are associated with an increase in depression-related symptomatology. We also reviewed two novel PPD-relevant rodent models based on postpartum adversity exposure that are characterized by mesolimbic DA downregulation. The first consists of deprivation of salient, species-expected social relationships (*via* pup removal), which precipitates an enduring negative affect state in the rat dam involving passive stress coping, social anhedonia, and DA hypofunction. The second one consists of creating an impoverished home cage environment, which stresses the dam, disrupts mother-infant interactions, dampens maternal motivation, increases passive stress coping, and blunts DA activity. In sum, pup removal and scarcity-adversity are emerging rodent models useful for studying adversity-induced changes in the maternal brain relevant to PPD while also providing support for the possibility that VTA DA neurons (and the mesolimbic DA system) may represent a therapeutic target for ameliorating reward-related deficits in PPD. The presented body of work provides ample opportunities for future directions examining neurobiological mechanisms contributing to PPD as well as potential treatments and/or interventions to ameliorate the negative impact of PPD on the mother-infant dyad, which may lead to enhanced mother and infant outcomes.

**Table 1:** DA system dysregulation in rodent models relevant to PPD.

**Model**	**Mesolimbic DA system effects**	**References**
Gestational stress	↓ length, branching, and spine density on NAc medium spiny neurons	Haim et al. (2014, 2016)
Pup removal	↓ VTA DA neuron activity	Rincón-Cortés and Grace (2021)
Scarcity-adversity	↓ VTA DA neuron activity	Rincón-Cortés and Grace (2022)
WKY rats	↓ striatal DA levels	Winokur et al. (2019)
FSL rats	↓ pup induced NAc DA release	Lavi-Avnon et al. (2008)
BALB/c mice	↓ striatal DA levels	Avraham et al. (2017)
Western (high fat) diet	↓ hippocampal and PFC DA metabolites ↓ hippocampal DA D2 receptor expression	Bolton et al. (2017)

↓ indicates reduction. PPD, postpartum depression; NAc, nucleus accumbens; VTA, ventral tegmental area; DA, dopamine; WKY, Wistar-Kyoto; FSL, Flinders Sensitive Line; PFC, prefrontal cortex.

## Data Availability Statement

The original contributions presented in the study are included in the article, further inquiries can be directed to the corresponding author.

## Author Contributions

MR-C conceived the idea for the manuscript and wrote the manuscript. AAG revised and provided feedback for the manuscript. MR-C and AAG edited and approved the manuscript for publication. All authors contributed to the article and approved the submitted version.
